# Estrogen maintains mitochondrial content and function in the right ventricle of rats with pulmonary hypertension

**DOI:** 10.14814/phy2.13157

**Published:** 2017-03-21

**Authors:** Aiping Liu, Jennifer Philip, Kalyan C. Vinnakota, Francoise Van den Bergh, Diana M. Tabima, Timothy Hacker, Daniel A. Beard, Naomi C. Chesler

**Affiliations:** ^1^ Department of Biomedical EngineeringUniversity of Wisconsin‐MadisonMadisonWisconsin; ^2^ Department of Molecular & Integrative PhysiologyUniversity of MichiganAnn ArborMichigan; ^3^ Department of MedicineUniversity of Wisconsin‐MadisonMadisonWisconsin

**Keywords:** Cardiopulmonary hemodynamics, mitochondria, sex differences

## Abstract

The typical cause of death in pulmonary hypertension (PH) is right ventricular (RV) failure, with females showing better survival rates than males. Recently, metabolic shift and mitochondrial dysfunction have been demonstrated in RV failure secondary to PH. In light of evidence showing that estrogen protects mitochondrial function and biogenesis in noncardiovascular systems, we hypothesized that the mechanism by which estrogen preserves RV function is via protection of mitochondrial content and oxidative capacity in PH. We used a well‐established model of PH (Sugen+Hypoxia) in ovariectomized female rats with/without estrogen treatment. RV functional measures were derived from pressure–volume relationships measured via RV catheterization in live rats. Citrate synthase activity, a marker of mitochondrial density, was measured in both RV and LV tissues. Respiratory capacity of mitochondria isolated from RV was measured using oxygraphy. We found that RV ventricular‐vascular coupling efficiency decreased in the placebo‐treated SuHx rats (0.78 ± 0.10 vs. 1.50 ± 0.13 in control, *P* < 0.05), whereas estrogen restored it. Mitochondrial density decreased in placebo‐treated SuHx rats (0.12 ± 0.01 vs. 0.15 ± 0.01 U citrate synthase/mg in control, *P* < 0.05), and estrogen attenuated the decrease. Mitochondrial quality and oxidative capacity tended to be lower in placebo‐treated SuHx rats only. The changes in mitochondrial biogenesis and function paralleled the expression levels of PGC‐1*α* in RV. Our results suggest that estrogen protects RV function by preserving mitochondrial content and oxidative capacity. This provides a mechanism by which estrogen provides protection in female PH patients and paves the way to develop estrogen and its targets as a novel RV‐specific therapy for PH.

## Introduction

Pulmonary hypertension (PH) is a fast, progressive, fatal vascular disease of the lung, with a 3‐year mortality rate of 55%(Humbert et al. 2010). The major cause of death is right ventricular (RV) failure (Noordegraaf and Galiè 2011). Despite a higher incidence of PH in women than men, female PH patients have better RV function and a better survival rate than male patients (Kawut et al. 2011; Jacobs et al. 2014). Both clinical and animal studies have demonstrated that the female sex hormone estrogen prevents RV functional and structural deterioration in PH (Ventetuolo et al. 2011; Liu et al. 2014; Frump et al. 2015). Our recent study showed that estrogen has a direct inotropic effect on the RV in addition to reducing RV afterload (Liu et al. 2014). However, the underlying mechanisms by which estrogen protects the RV in PH remain unclear, which limits the therapeutic potential of estrogen‐based RV‐specific therapies in PH.

To function, the heart needs a continuous supply of chemical energy (or ATP), 95% of which comes from phosphorylation oxidation of the mitochondria (Doenst et al. 2013). Akin to left ventricle failure secondary to systemic hypertension (Sabbah et al. 1992; Sharov et al. 2000), RV failure secondary to PH has been recently characterized by abnormal energy metabolism and mitochondrial dysfunction (Piao et al. 2010b; Gomez‐Arroyo et al. 2013). In adaption to increased afterload in PH, the RV exhibits abnormal mitochondrial oxidation by metabolically shifting from glucose oxidation to glycolysis (Oikawa et al. 2005; Lundgrin et al. 2013), which is believed to underlie RV dysfunction because modulation of this metabolic shift improves RV function (Piao et al. 2010a). Dysfunctional RVs in both humans and rats have demonstrated impaired mitochondria structure and respiration (Gomez‐Arroyo et al. 2013), consistent with a down‐regulation of PGC‐1*α* and its downstream target genes, which are involved in mitochondrial biogenesis and oxidative capacity (Liang and Ward 2006; Ventura‐Clapier et al. 2008).

Estrogen is well known to modulate mitochondrial biogenesis and function in brain and cancer cells (Klinge 2008), but few studies have investigated the effects of estrogen on mitochondria in the cardiovascular system. In rodent hearts with ischemia‐reperfusion injury, estrogen‐treated animals have higher mitochondrial complex IV expression (Hsieh et al. 2006b), less mitochondrial damage, and greater maintenance of mitochondrial function (Zhai et al. 2000) compared to the nontreated animals. The direct protective effects of estrogen on mitochondrial structure and function have been associated with improved cardiac function in these rodent hearts with ischemia‐reperfusion injury (Zhai et al. 2000; Hsieh et al. 2006b). However, it remains unknown whether the protection of RV function by estrogen is linked to its protection of mitochondrial remodeling in PH.

Here, we hypothesized that estrogen preserves RV mitochondrial content and oxidative capacity, which protects the RV from dysfunction in severe PH. We used the sugen/hypoxia (SuHx) model of PH in rats (Abe et al. 2010), which recapitulates angio‐obliterative features of human PH. Female rats were ovariectomized and some were supplemented with a physiological level of estrogen. We measured RV pressure‐volume loops in vivo via RV catheterization, from which we derived RV function measures. In addition, we measured RV mitochondrial content and respiration of isolated RV mitochondria. We found that RV metabolic remodeling precedes RV systolic dysfunction and that the protective effect of estrogen on RV function is associated with preservation of mitochondrial density and function in PH. This study for the first time links the protection of estrogen on metabolic remodeling to RV functional adaption in PH and underscores the potential of estrogen as a RV‐specific therapy.

## Methods

### Animal care and treatment

Female Sprague‐Dawley rats, 4–5 weeks old, were ovariectomized (OVX) by a commercial vendor (Harland Laboratory) to eliminate natural fluctuations in estrogen levels. After 1 week, to ensure depletion of endogenous estrogen stores, rats were implanted subcutaneously with either estradiol‐17 pellets (75 *μ*g/kg/day (Lahm et al. 2012); Innovation Research of America) or placebo pellets as control for the effects of estrogen. Immediately following the pellet implantation to induce pulmonary hypertension, rats from both estrogen and placebo groups were injected intraperitoneally with the VEGF receptor inhibitor SU5416 at 20 mg/kg and exposed to normobaric hypoxia (10% O_2_) for 4 weeks (SuHx), followed by 10 weeks of normoxia. Control rats from both estrogen and placebo groups received no SU5416 and were kept in room air conditions for 14 weeks. All rats were housed at room temperature with a 12‐hour dark/light cycle and fed a copper‐supplemented soy‐free rodent diet (TD.130197, Harland) with free access to water. Of these animals, one group of rats (SuHx and control, estrogen and placebo‐treated, *n = *10–11 each) was used for echocardiography and RV pressure and volume measurements; terminal experiments were performed at the University of Wisconsin‐Madison. A second group of rats (SuHx and control, estrogen and placebo‐treated; *n = *6 each) was used for RV bioenergetic measurements; terminal experiments were performed at the University of Michigan. All procedures were approved by the University of Wisconsin‐Madison and University of Michigan Institutional Animal Care and Use Committees.

### Hemodynamics

#### Echocardiography

In the first group of rats, transthoracic echocardiography was performed to examine right ventricle (RV) morphology and function in vivo. As described previously (Markandeya et al. 2016), rats were ventilated with 1% isofluorane 99% O_2_ and maintained on a heated platform. RV wall thickness was obtained from M‐ mode images using the leading edge‐to‐leading edge convention (Visual Sonics Vevo 770 ultrasonography with a 17‐MHz transducer). Pulmonary flow was measured at the level of the pulmonary valve using Doppler imaging. The isovolumic relaxation time and PA ejection time, the reference parameters for RV diastolic and systolic function, respectively, were derived from the pulmonary flow images as described (Lindqvist et al. 2005). All parameters were measured over at least three consecutive cardiac cycles and averaged. Stroke volume (SV) was calculated as VTI × π*D* ^2^/4, where VTI is the time integral of flow velocity, and D is the PA diameter at the site where the flow was measured, and obtained at diastole in B‐mode images. Cardiac output (CO) was calculated as SV × heart rate. Rats were allowed to recover before pressure and volume measurements were obtained via right heart catheterization.

#### Right heart catheterization

RV pressure and volume (PV) were measured simultaneously following the procedures described by Liu et al. (2014). Briefly, rats were anesthetized with urethane (2.0 mg/kg), intubated, and ventilated at room air with a tidal volume of 2–2.5 mL and a respiratory rate of 80–100 breaths/min to minimize the impact of open‐chest surgery on the heart rate and respiration of the animal (Schreier et al. 2014). The animal was placed supine on a heated pad to keep the body temperature at ~37°C. After a tracheotomy was performed, a 1.9 F variable segment admittance PV catheter was inserted into the RV through the apex to acquire PV loops. Signals were recorded at 1000 Hz. After baseline recordings, a brief vena cava occlusion was performed to alter venous return. Systemic pressure was monitored at the aortic arch via the right carotid artery.

RV function was quantified using well‐established parameters derived from baseline RV PV loops, including RV end‐systolic pressure (P_es_), SV, CO, ejection fraction, RV maximal and minimal derivative of pressure (dP/d*t*_max_ and dP/d*t*_min_), and chamber compliance (SV/RV PP). To account for hypertension and estrogen‐dependent weight changes, cardiac index (CI) was calculated as CO normalized by body weight (BW). Load‐independent indices of systolic function such as end‐systolic elastance (E_es_) were derived from PV loops with vena cava occlusion. To assess RV afterload and ventricular‐vascular coupling efficiency, effective arterial elastance (E_a_), and E_es_/E_a_ were calculated (Kelly et al. 1992; Santamore and Dell'Italia 1998). Cardiac energetics were assessed via pressure‐volume area (PVA; an estimation of myocardial oxygen consumption), external work, and ventricular mechanical efficiency derived from the baseline RV PV relations as previously established (Nozawa et al. 1988).

### Bioenergetics

#### Tissue preparation

After induction of anesthsia with intraperitoneal injection of ketamine (90 mg/kg) and dexmedetomidine (0.5 mg/kg) followed by 0.5 mL heparin (1000 USP units/mL), the heart was cannulated in situ to perfuse the heart with ice‐cold cardioplegia solution (5 mmol/L KCl, 100 mmol/L NaCl, 10 mmol/L Dextrose, 25 mmol/L MOPS, 1 mmol/L EGTA, pH 7.2) for 5 min in order to remove residual blood. The RV and LV free walls, were then quickly dissected, blotted, and weighed. Tissue samples were used for mitochondrial isolation (~150 mg) and mitochondrial content determination through citrate synthase activity assay (~10 mg).

#### Mitochondrial isolation

Because myocytes consists of 75% of heart volume (Vliegen et al. 1991), the majority of mitochondria isolated from heart tissues were from myocytes. The tissue samples for mitochondrial isolation were minced and homogenized in ice‐cold isolation buffer (200 mmol/L mannitol, 50 mmol/L sucrose, 5 mmol/L KH2PO4, 5 mmol/L MOPS, 1 mmol/L EGTA, 0.1% BSA, pH 7.2) containing protease solution (5 U/mL in 2.5 mL finally diluated to 25 mL) with a hand‐hold CAT ×120 homogenizer. RV mitochondria were isolated using differential centrifugation as described previously (Vinnakota et al. 2011). Briefly, the homogenate was twice centrifuged (Eppendorf F‐34‐6‐38 rotor and 5810R centrifuge) under 4°C at high speed i.e., 8000 rcf for 10 min to remove the protease. The supernatant was discarded and the pellets were resuspended in isolation buffer and centrifuged at 700 rcf to remove other cellular debris. The supernatant was centrifuged again at 8000 rcf to yield mitochondrial pellets. The mitochondrial pellets were resuspended in a small volume of isolation buffer and stored on ice for the duration of the experiment.

#### Citrate synthase activity

Citrate synthase (CS) activity, a marker of mitochondrial density, was measured in both RV and LV tissues, as well as in the mitochondrial suspension, polarographically in a spectrometer as previously described (Eigentler et al. 2012). Heart tissues were minced and homogenized in 0.1mol/L Tris (pH7) with a hand‐hold CAT × 120 homogenizer for 20 sec before CS activity measurement. A mitochondrial concentration equivalent to 0.337 units of CS activity/mL (which corresponds to ∼0.1 mg protein/mL in a Bradford assay calibrated against bovine serum albumin standards) was used in mitochondrial respiration.

#### Oxygraphy

Oxygen consumption was measured at 37°C using high resolution Oroboros oxygraphy (Oxygraph 2K, Oroboros Instruments, Innsbruck, Austria), as described in (Vinnakota et al. 2016). Briefly, respiration was measured in respiration buffer (107.5 mmol/L KCl, 5 mmol/L K2HPO4, 50 mmol/L MOPS, 1 mmol/L EGTA, 1.5 mmol/L MgCl2, and 0.1% w/w BSA essentially fatty acid free) pH to 7.2 at 37°C. The amount of mitochondria used was determined by their CS activity (337 milli‐units CS per ml of respiration buffer in the oxygraph chamber – 2 mL total).

The following respiratory parameters were measured in order: (Abe et al. 2010) Basal respiratory rate (V_0_), respiratory rate (V_sub_) after addition of substrates (State 2 respiration); and maximal respiratory rate (V_max_) after addition of ADP 500 *μ*mol/L final (state 3 respiration). The functional integrity of the mitochondria was assessed by the respiratory control index defined here as V_max_/V_sub_(state 3/state 2 respiration). Two combinations of substrates were used: 1 mmol/L malate and 5 mmol/L pyruvate or 20 *μ*mol/L palmitoyl‐L‐carnitine.

The P/O ratio, the ratio of rate of ATP synthesis to rate of oxygen atom consumption, was determined for respiration using carbohydrate and fatty acid substrates. Briefly, 674 milli‐units CS equivalent of mitochondria suspension and substrates (2 mmol/L malate and 350 *μ*mol/L pyruvate, or 2 mmol/L malate and 20 *μ*mol/L palmitoyl‐L‐carnitine) were sequentially added into 2 mL of respiration buffer as described above. After 2 min of incubation to establish an energized mitochondrial state 2, as solution of 250 *μ*mol/L ADP was infused into the oxygraphic media at a rate of 0.026 *μ*l/s for 380 sec. The P/O ratio was determined as the ratio between the rate of ADP infusion and the measured rate of oxygen atom consumed.

#### Protein immunoblotting

Right ventricular tissues were homogenized and lysed in RIPA buffer (25 mmol/L Tris•HCl pH 7.6, 150 mmol/L NaCl, 1% NP‐40, 1% sodium deoxycholate, 0.1% SDS) plus 1:100 HALT protease and phosphatase inhibitor cocktail (Thermo Scientific, Rockford, IL). Equal amounts of protein for each sample were separated by SDS‐PAGE, transferred onto a nitrocellulose membrane, and immunoblotted. Anti‐PGC1‐*α* primary antibody (Santa Cruz Biotechnology, Santa Cruz, CA) was used at a dilution of 1:500. Anti‐Actin primary antibody (Santa Cruz Biotechnology, Santa Cruz, CA) was used as a loading control. Bands were visualized with ECL Western blotting substrate (Thermo Scientific, Rockford, IL). Band intensity was quantitated using NIH ImageJ software.

### Statistical analysis

All results are presented as mean ± standard error. The significance of the changes in RV function and bioenergetics with estrogen treatment and SuHx exposure was assessed with two‐way analysis of variance (estrogen vs. placebo and SuHx vs. control) followed with Tukey multiple comparisons at a significance level of 0.05. Statistical analysis was performed using R software (Foundation for statistical computing, version 2.5.1).

## Results

### Estrogen attenuated RV structural remodeling in rats with PH

As estrogen significantly influenced the body size and weight of the animals (Table 1), normalized right ventricular remodeling indices (RV/BW, RV/LVS and RV/TL) were used to assess RV hypertrophy. All these measures significantly increased in the placebo‐treated SuHx group, indicative of RV hypertrophy in PH. Estrogen treatment attenuated the increase in RV/LVS and RV/TL in the SuHx group. Consistent with this result, the RV wall thickness measured using echocardiography significantly increased in the placebo‐treated SuHx group, but not in the estrogen‐treated SuHx group (Table 2).

**Table 1:** Body weight and morphological parameters of right and left ventricles

	Control_Placebo	Control_Estrogen	SuHx_Placebo	SuHx_Estrogen
BW, g	335.3 ± 6.5	219.0 ± 10.1^b^	326.2 ± 5.8	212.2 ± 9.7^b^
RV, mg	172.4 ± 4.5	148.9 ± 6.2	284.7 ± 22.4^a^	167.6 ± 6.5^b^
LVS, mg	684.5 ± 16.0	544.5 ± 23.7^b^	746.5 ± 30.7	546.4 ± 21.0^b^
TL, mm	40.0 ± 0.4	34.5 ± 1.2^b^	39.9 ± 0.3	35.8 ± 0.6^b^
RV/BW, mg/g	0.52 ± 0.01	0.67 ± 0.02	0.87 ± 0.07^a^	0.82 ± 0.05
LVS/BW, mg/g	2.05 ± 0.04	2.44 ± 0.06^b^	2.29 ± 0.09	2.63 ± 0.09^b^
RV/LVS, mg/mg	0.252 ± 0.004	0.28 ± 0.01	0.38 ± 0.02^a^	0.31 ± 0.02^b^
RV/TL, mg/mm	4.3 ± 0.1	4.2 ± 0.2	7.1 ± 0.5^a^	4.7 ± 0.2^b^

Data = mean ± SE. BW, body weight; RV, right ventricle; LVS, left ventricle plus septum; TL, tibia bone length.

a *P* < 0.05 versus Control;

b *P* < 0.05 versus Placebo. *n* = 10–11 in all groups.

**Table 2:** Morphological and hemodynamic parameters measured using echocardiography in live rats

	Control_Placebo	Control_Estrogen	SuHx_Placebo	SuHx_Estrogen
h, mm	0.62 ± 0.06	0.61 ± 0.05	0.86 ± 0.04^a^	0.76 ± 0.03
SV, *μ*L	214.7 ± 9.8	184.8 ± 12.8	208.0 ± 10.1	176.2 ± 12.2
CO, mL/min	71.5 ± 3.9	63.9 ± 4.9	69.3 ± 4.6	60.7 ± 5.5
CI, mL/min/kg	213.6 ± 10.4	311.0 ± 23.7^b^	212.3 ± 12.7	288.0 ± 20.3
IVRT, ms	29.4 ± 1.2	24.1 ± 1.6	34.7 ± 1.7	29.7 ± 1.7^a^
ET, ms	77.2 ± 2.9	68.2 ± 2.4	67.6 ± 1.5	54.8 ± 4.9^a^

Data = mean ± SE. h, ventricular wall thickness; SV, stroke volume; CO, cardiac output; CI, cardiac index; IVRT, isovolumic relaxation time; ET, ejection time.

a *P* < 0.05 versus Control;

b *P* < 0.05 versus Placebo. *n* = 7–10 in all groups.

### Estrogen protected RV function and mechanical efficiency in rats with PH

**Figure 1:**
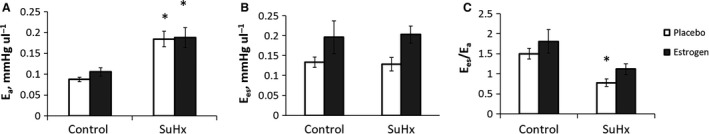
Estrogen protects right ventricular‐vascular coupling in SuHx‐induced PH. (A) Arterial elastance, E_a_; (B) end‐systolic elastance, E_es_; and (C) ventricular‐vascular coupling efficiency, E_es_/E_a_. *, *P* < 0.05 versus Control, #, *P* < 0.05 versus Placebo. *n* = 9–11 in all groups.

SuHx significantly increased pulmonary P_es_ in rats (Table 3), indicating successful creation of PH. E_a_, an index for RV afterload, significantly increased in both placebo‐ and estrogen‐treated SuHx rats to a similar level (Fig. 1). However, RV E_es_, an index for load‐independent ventricular contractility, did not increase with E_a_ in the placebo‐treated SuHx group, resulting in a significant reduction of ventricular‐vascular coupling efficiency (E_es_/E_a_). As estrogen treatment tended to improve RV contractility (although no statistical significance was detected), the ventricular‐vascular coupling efficiency was maintained in the estrogen‐treated SuHx group. While neither SV nor CO was affected by SuHx exposure or estrogen treatment, CI was higher in the estrogen‐treated rats compared to the placebo‐treated rats (Table 3). SV/RV PP, an index for ventricular compliance, decreased in both placebo‐ and estrogen‐treated SuHx groups, indicative of stiffer RV in PH. The increase in ventricular stiffness is consistent with an increase in the isovolumic relaxation time, an echocardiographic measure of RV diastolic dysfunction (Table 2).

**Table 3:** Hemodynamic parameters measured using right heart catheterization in live rats

	Control_Placebo	Control_Estrogen	SuHx_Placebo	SuHx_Estrogen
sys P_s_, mmHg	112.8 ± 6.9	110.4 ± 6.0	132.6 ± 5.1	119 ± 6
P_es_, mmHg	24.2 ± 1.1	24.7 ± 1.4	45.6 ± 5.6^a^	40.6 ± 3.7^a^
dP/dt _max_, mmHg/s	2719 ± 168	2385 ± 219	4066 ± 262^a^	3273 ± 310
dP/dt _min_, mmHg/s	−2064 ± 137	−1857 ± 196	−3426 ± 290^a^	−2664 ± 460
EF, %	56 ± 3	63 ± 3	54 ± 3	52 ± 5
HR, bpm	350 ± 10	329 ± 18.7	341 ± 13	338 ± 17
SV, *μ*L	283.5 ± 15.5	245.6 ± 17.3	246.8 ± 14.6	226.5 ± 16.2
CO, mL/min	98.7 ± 4.9	80.5 ± 7.0	83.2 ± 3.8	76.2 ± 6.1
CI, mL/min/kg	293.3 ± 14.0	380.9 ± 26.5^b^	252.4 ± 8.6	360.9 ± 22.8^b^
SV/RV PP, *μ*L/mmHg	13.3 ± 1.0	11.8 ± 1.0	6.3 ± 0.5^a^	6.4 ± 0.7^a^
H_ct_, %	49 ± 1	42 ± 1^b^	48 ± 1	41 ± 2

Data = mean ± SE. sys P_s_, peak systemic pressure; P_es_, end‐systolic pulmonary pressure; dP/dt _max_ and dP/dt _min_, maximal and minimal pressure gradient; HR, heart rate; SV, stroke volume; CO, cardiac output; CI, cardiac index; SV/RV PP, ventricular compliance; Hct, hematocrit.

a *P* < 0.05 versus Control;

b *P* < 0.05 versus Placebo. *n* = 9‐11 in all groups.

**Figure 2:**
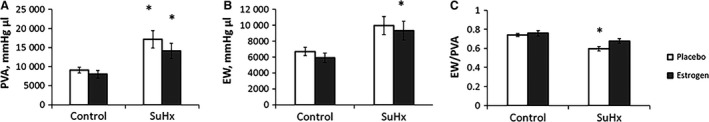
Estrogen protects RV mechanical efficiency in SuHx‐induced PH. (A) pressure‐volume area, PVA; (B) External work, EW; and (C) mechanical efficiency, EW/PVA. *, *P* < 0.05 versus Control, #, *P* < 0.05 versus Placebo. *n* = 9–11 in all groups.

The total mechanical energy generated by ventricular contraction represented by PVA significantly increased in both SuHx groups (Fig. 2A). The effective energy output (EW) significantly increased in the estrogen‐treated SuHx group but did not change in the placebo‐treated SuHx group (Fig. 2B). As a result, the cardiac mechanical efficiency (EW/PVA) decreased in the placebo‐treated SuHx group, whereas mechanical efficiency was preserved in the estrogen‐treated SuHx group (Fig. 2C).

### Estrogen protected RV metabolic remodeling in rats with PH

**Figure 3:**
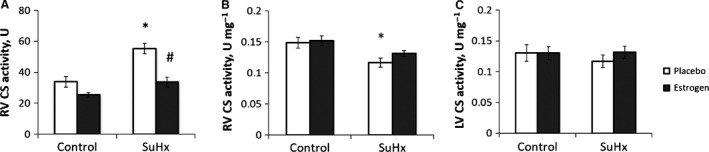
Estrogen protects citrate RV synthase (CS) activity in SuHx‐induced PH. (A) RV CS activity normalized by RV weight; (B) CS activity of the entire RV; and (C) LV CS activity normalized by LV weight. *, *P* < 0.05 versus Control, #, *P* < 0.05 versus Placebo. *n* = 4–6 in all groups.

**Figure 4:**
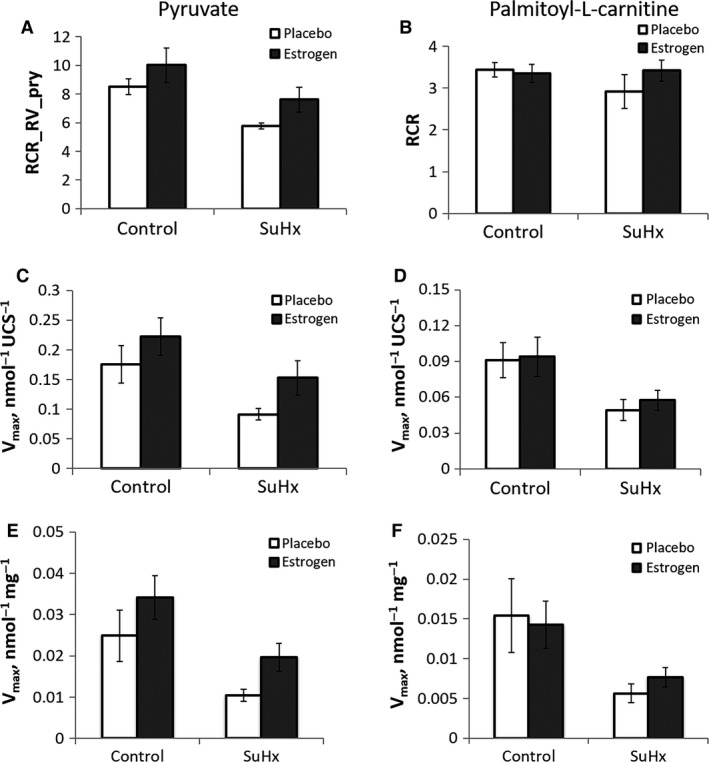
Effects of estrogen on RV mitochondrial quality and oxidative capacity in SuHx‐induced PH. Respiratory control ratio, RCR in substrates pyruvate (A) and palmitoyl‐L‐carnitine (B); Mitochondrial maximal oxygen consumption rate (V_max_) of state 3 in substrates pyruvate (C) and palmitoyl‐L‐carnitine (D); RV tissue respiration capacity in substrates pyruvate (E) and palmitoyl‐L‐carnitine (F). *n* = 4–6 in all groups.

**Figure 5:**
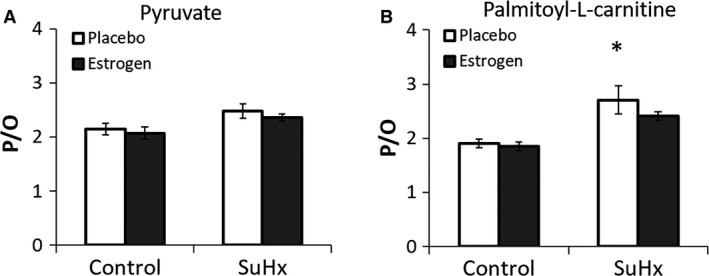
RV mitochondrial P/O ratio measured in pyruvate (A) and palmitoyl‐L‐carnitine (B). *, *P* < 0.05 versus Control, #, *P* < 0.05 versus Placebo. *n* = 4–6 in all groups.

The total CS activity of the entire RV was significantly higher in the placebo‐treated SuHx group than in the control and estrogen‐treated SuHx groups (Fig. 3A), due primarily to the fact that the total mass of RV tissue was significantly elevated in this group compared to the others. However, CS activity measured per unit mass of RV tissue was significantly lower in the placebo‐treated SuHx group, suggesting a decrease in mitochondrial density. Estrogen treatment attenuated the decrease in the normalized CS activity and thus acted to maintain the mitochondrial density in PH (Fig. 3B). CS activity in LV did not change with either SuHx or estrogen treatment (Fig. 3C). The RCR, a measure of mitochondrial coupling, and V_max,_ a measure of mitochondrial respiration capacity, tended to be lower in both pyruvate and palmitoyl‐L‐carnitine as the substrate in the placebo‐treated SuHx group compared to the control (Fig. 4A–D). Estrogen treatment attenuated the decrease in RCR and V_max_, suggesting that estrogen protected against damage to the quality and respiration capability of RV mitochondria in PH. Respiration capacity of RV tissue, defined as V_max_ multiplied by the CS activity per unit mass of RV, followed the same trend as the mitochondrial respiration capacity (Fig. 4E and F). The P/O ratio, a measure of mitochondrial respiratory efficiency, was significantly higher in the substrate palmitoyl‐L‐carnitine in the placebo‐treated SuHx group compared to the control group, and estrogen had limited effect on the P/O ratio in both substrates (Fig. 5).

**Figure 6:**
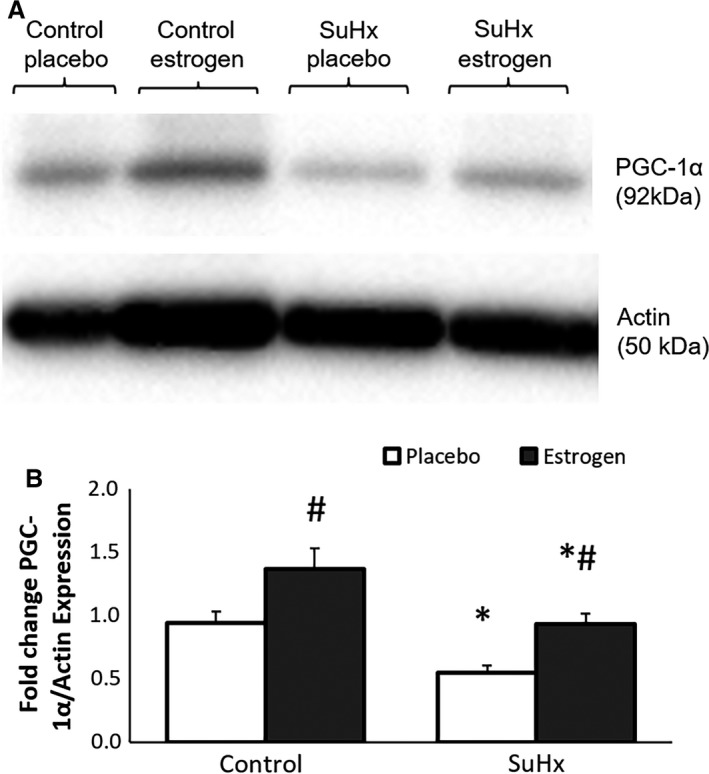
Representative immunoblot (upper panel) showing PGC‐1*α* expression for control and SuHx rat RV with placebo or estrogen treatment. Actin was used as a loading control. Densitometric analysis shown below. *, *P* < 0.05 versus Control, #, *P* < 0.05 versus Placebo. *n* = 5–6 in all groups.

PGC‐1*α* is known to be a key regulator of oxidative metabolism and mitochondrial function and has recently been shown to be down‐regulated in the setting of SuHx‐induced PH and RV failure (Gomez‐Arroyo et al. 2013). Given our findings of decreased mitochondrial density and oxidative capacity in the setting of PH, which was restored by estrogen treatment, we examined the effect of estrogen treatment on PGC‐1*α*. As demonstrated by Fig. 6, there is an over 40% decrease in PGC‐1*α* expression in the RV in the setting of PH. Estrogen treatment led to a significant increase in PGC‐1*α* expression under control conditions. Additionally, estrogen treatment led to the preservation of PGC‐1*α* levels at near control levels with PH. Consistent with our findings of estrogen's protective effects on mitochondrial density and oxidative capacity, this result indicates that increasing PGC‐1*α* levels is one mechanism by which estrogen preserves RV metabolism and function in the setting of PH.

## Discussion

In this study, we investigated the metabolic mechanisms behind the protection of estrogen on RV function in PH. We revealed for the first time that estrogen preserved RV ventricular‐vascular efficiency by enhancing RV contractility reserve with limited effects on the afterload in a rat model of PH. We also demonstrated decreases in RV mitochondrial density and function with an increase in afterload that did not occur in the presence of estrogen. These results suggest that the protective effects of estrogen on RV function are linked to metabolism. We believe that our findings will contribute to an improved understanding of RV failure and more effective therapies to combat this devastating disease.

To resolve sex disparities in PH outcomes, researchers have focused on the roles of the female sex hormone estrogen in RV adaptation and demonstrated that estrogen limits RV functional and structural remodeling (Nadadur et al. 2012; Frump et al. 2015). For example, Frump and colleagues showed that estrogen repletion ameliorated SuHx‐induced alterations in RV hemodynamics and protected RV exercise capacity (Frump et al. 2015). Nadadur et al. (2012) showed that estrogen therapy reversed RV remodeling and prevented RV progression to failure in rats treated with monocrotaline. While intriguing, these studies lack thorough mechanical measurements of RV function and the interaction between the RV and pulmonary vascular bed, which makes it difficult to evaluate whether estrogen improves RV performance through direct (i.e., enhancing myocardial contraction) or indirect (i.e., attenuating pulmonary vascular remodeling) means. Using PV loops from RV catheterization in live mice, we previously revealed that estrogen has a direct inotropic effect on the RV and also attenuates the increase in RV afterload in an early, mild stage of PH (Liu et al. 2014). However, as PH progresses, the mechanisms of estrogenic protection on the RV may evolve. In this study, using a well‐established model of a late, severe stage of PH in rats, we revealed for the first time that estrogen enhanced RV contractility with limited effects on RV afterload, resulting in preserved ventricular‐vascular coupling efficiency. Estrogen enhanced CI and E_es_ in both control and diseased animals compared to the placebo‐treated animals, similar to the previous findings (Frump et al. 2015; Lahm et al.^2016^), indicating that estrogen mediates superior adaption of RV function to PH. Interestingly, this enhancement in RV function did not result in additional energy demands because estrogen preserved mechanical efficiency.

It is also worth noting that the SuHx exposure in our study did not create the same level of severity as previously reported (de Raaf et al. 2014; Toba et al. 2014; Frump et al. 2015), which can be attributed to different SuHx/Normoxia protocols, different dosage of Sugen, and different strains or colonies of rats. It has been reported that SuHx‐induced increase in RV systolic pressure regressed after the rats returned to normoxia (de Raaf et al. 2014). Variation in the strains of rats, even between colonies of the same strain has a remarkable influence on the response to SuHx (Jiang et al. 2016). In this study, the prolonged exposure to normoxia and the rat colony likely explain the less severe phenotype of PH compared to the other studies. Despite the fact that no animals exhibited overt RV failure (i.e., CO and EF were preserved in all animals), the decrease in ventricular‐vascular coupling efficiency in the placebo‐treated diseased animals suggests a transition of RV function from a compensated state to a decompensated state (Wang et al. 2013).

Cardiac mitochondria are the powerhouse of the heart; and mitochondrial abnormalities play a pivotal role in the development of heart failure (Bayeva et al. 2013). In the hypertrophied and failing RV secondary to PH, earlier studies identified mitochondrial hyperpolarization (Nagendran et al. 2008), increased reactive oxygen species (ROS) production (Redout et al. 2007), mitochondrial structural and functional abnormalities (Nouette‐Gaulain et al. 2005; Gomez‐Arroyo et al. 2013), and a metabolic shift from fatty acid oxidation to glycolysis (Piao et al. 2010b). Drugs targeting some of these mitochondrial derangements have been shown to improve RV function (Mouchaers et al. 2009; Piao et al. 2010a; Dumas Roque La et al. 2012), which links RV metabolic remodeling and mechanical function in PH. In this study, we sought to reveal the mitochondrial basis of estrogenic protection on RV adaptation to the persistently increased afterload in PH. Similar to a previous study in male rats (Gomez‐Arroyo et al. 2013), we found that in estrogen‐deficient (OVX) female rats, mitochondrial volume density in RV but not in LV decreased while the total number of mitochondria in RV increased, indicating that the rate of mitochondria biogenesis could not keep pace with the rate of tissue growth in response to increased afterload. This decrease in mitochondria volume density was paralleled by the reduced expression of PGC‐1*α*, the master regulator of mitochondrial biogenesis (Scarpulla 2011), suggesting that the decreased mitochondrial density was a result of the defects in the translational pathway in mitochondria biogenesis. Moreover, we found that estrogen repletion preserved RV PGC‐1*α* and mitochondrial content. This finding is profound because animal studies on LV failure suggest that impaired mitochondrial biogenesis due to down‐regulation of PGC‐1*α* transcriptional pathway is integral to mitochondrial dysfunction (Ventura‐Clapier et al. 2008). The preservation of mitochondrial biogenesis underlies the protective effects of estrogen on RV function and provides a mechanistic basis for estrogen as a novel RV‐specific therapy in PH. The abilities of estrogen to preserve mitochondrial biogenesis and PGC‐1*α* expression have been also documented in the trauma‐hemorrhage mouse heart model (Hsieh et al. 2005, 2006a).

Besides reduced mitochondrial density, mitochondrial quality measured with RCR and ADP‐stimulated oxidation capability tended to be depressed in the hypertrophied RV. The low RCR, a quality measure for mitochondrial preparation, in the placebo‐treated SuHx rats implies that either the RV mitochondria in these animals were damaged or prone to be damaged even though the isolation procedures for all the animals were the same. This finding is consistent with the observations of abnormalities in ultrastructure of RV mitochondria and ROS‐induced mtDNA damage in the dysfunctional RV tissues in male rats with SuHx‐induced severe PH (Gomez‐Arroyo et al. 2013). The reduction of mitochondrial oxygen consumption rate in RV myocytes was also reported in monocrotaline‐induced RV dysfunction, and the reduction of mitochondrial oxidative capacity was inversely related to the decrease in the index for RV function measured with echocardiography (Daicho et al. 2009). Our finding that estrogen attenuated the alterations in mitochondria quality and oxidation capacity further supports the involvement of mitochondria in the estrogenic protection of RV function. In the PH‐induced failing RV, failure is associated with increased ROS production (Redout et al. 2007), which is known to cause mitochondrial structural and functional damage. The antioxidant properties of estrogen may protect myocardial mitochondria against oxidative damage (Persky et al. 2000; Borrás et al. 2010).

Surprisingly, the P/O ratio, an index for respiration efficiency, increased in the substrate palmitoyl‐L‐carnitine in the placebo‐treated SuHx rats, indicating lower oxygen consumption per unit of ADP provided in fatty acid oxidation and estrogen had limited effects on the P/O ratio. The P/O ratio in the substrate pyruvate followed a similar trend. The SuHx‐induced increase in respiration efficiency is opposite to the finding by Drake et al. (2013), who showed that P/O ratio was reduced in male SuHx rats. We speculate that the different findings in the P/O ratio are attributable to different stages of RV dysfunction and/or sex differences in mitochondrial accommodation to pressure‐overload (Witt et al. 2008). The increase in efficiency of oxygen consumption likely represents another compensatory mechanism besides the metabolic switch to meet the increasing energy demand.

### Limitations

We used different cohorts of animals to conduct the mechanical and metabolism studies to preserve tissue quality for the latter. To minimize the differences between the two cohorts, we tightly controlled the animal colony, age, feed, and treatment to be the same. Also, RV mitochondria were isolated and therefore not tested in their in vivo environment, which is known to affect mitochondrial function and can be altered by RV remodeling (Wu et al. 2009; Ryan and Archer 2014). Since all the preparation procedures for mitochondria were kept the same for all the experimental groups, we expect the trends would hold or be further amplified in their physiological conditions. Further, our SuHx protocol did not create PH as severe as prior reports and did not create RV failure; thus, the protection of estrogen on RV function and mitochondrial remodeling were not as pronounced as would be expected in the failing RV. However, our model captured indicators of a transition from a compensated RV to a decompensated RV and suggests that mitochondrial remodeling may occur prior to RV failure.

## Conclusions

Despite the significance of RV function to survival in PH, there are no therapies that directly and selectively improve RV function. Motivated by the clinical observation that female patients have superior RV functional adaption in PH, we investigated the metabolic mechanisms by which estrogen offers protection to RV function in PH. Our study for the first time reveals that estrogen preserves RV mitochondrial density and tends to preserve function in PH‐induced RV hypertrophy, which may underlie the estrogenic improvement of RV contractility and mechanical efficiency. If derangements in mitochondria occur early and precede the transition from RV dysfunction to failure, estrogen could be the basis of a novel RV‐specific therapy for PH patients.

## Conflict of Interest

None declared.
